# Targeting FRβ+ tumor associated macrophages with car T cells in ovarian cancer

**DOI:** 10.1186/2051-1426-3-S2-P32

**Published:** 2015-11-04

**Authors:** Rachel C Lynn, Takami Matsuyama, Daniel J Powell

**Affiliations:** 1 University of Pennsylvania, Philadelphia, PA, USA; 2 Department of Immunology, Graduate School of Medical and Dental Sciences, Kagoshima University, Kagoshima, Japan

## Background

Chimeric antigen receptor (CAR) T cell therapy has shown dramatic clinical success in CD19+ leukemia and lymphoma patients. However, CAR T cell therapy for epithelial cancers has been less successful. Developing effective CAR T cell therapies for other types of cancer may involve overcoming several obstacles associated with solid tumors. Poor blood supply and dense stromal components can hinder access of CAR T cells to tumor cells. In addition, immunosuppressive elements in the tumor microenvironment may suppress T cell functional activity. Tumor associated macrophages (TAMs) have been identified as key pro-tumor players in the microenvironment. Tumors co-opt macrophage function to help promote tumor growth (“M2” polarization) via many diverse mechanisms including promoting angiogenesis, metastasis, and immune evasion. Indeed, the presence of TAMs correlates with worse overall prognosis in many types of cancer, including ovarian cancer patients. We hypothesized that utilizing the power of CAR T cells to target TAMs could be an effective way to improve CAR T cell therapy in epithelial cancer.

## Methods

Folate receptor beta (FRβ), a GPI-linked membrane protein that mediates folic acid uptake, has previously been described on TAMs in human cancer patients and mouse models of cancer. We found that FRβ is highly expressed in monocyte-derived M2 polarized macrophages. Using high affinity human FRβ-specific CAR T cells (m923), we observed significant IFN-γ secretion and dose-dependent cytolysis of M2 polarized macrophages. When co-cultured with FRβ(-) human ovarian cancer cell line SKOV3, m923 CAR T cells mediated bystander killing of FRβ(-) tumor cells when FRβ+ M2 polarized macrophages were present. Next, we confirmed high expression of FRβ in TAMs from primary ovarian cancer. m923 CAR T cells displayed specific activation in the presence of primary ovarian cancer, supporting the hypothesis that m923 CAR T cells could be utilized to eliminate FRβ+ TAMs in patients.

To test our hypothesis in preclinical mouse models, we developed a mouse FRβ-specific CAR (CL10).

## Results

Using the implantable murine ovarian carcinoma ID8, we confirmed expression of FRβ in F4/80^+^/CD204^+^ TAMs isolated from peritoneal tumor ascites by flow cytometry. In addition, CL10 CAR T cells displayed significant specific reactivity against isolated CD11b^+^ TAMs. Our preliminary data suggest that adoptive transfer of CL10 CAR T cells into ID8 tumor-bearing mice results in destruction of FRβ+ TAMs and delayed tumor growth. Future experiments will determine whether combining CL10 with tumor-directed CAR T cells will result in synergistic anti-tumor effects.

## Conclusions

In conclusion, our results suggest targeting TAMs with CAR T cells may be a novel way to enhance CAR therapy for solid tumors.

